# Rhamnolipid-Coated Iron Oxide Nanoparticles as a Novel Multitarget Candidate against Major Foodborne E. coli Serotypes and Methicillin-Resistant S. aureus

**DOI:** 10.1128/spectrum.00250-22

**Published:** 2022-07-19

**Authors:** Mohamed Sharaf, Alaa H. Sewid, H. I. Hamouda, Mohamed G. Elharrif, Azza S. El-Demerdash, Afaf Alharthi, Nada Hashim, Anas Abdullah Hamad, Samy Selim, Dalal Hussien M. Alkhalifah, Wael N. Hozzein, Mohnad Abdalla, Taisir Saber

**Affiliations:** a Department of Biochemistry, Faculty of Agriculture, Al-Azhar University, Nasr City, Cairo, Egypt; b Department of Biochemistry and Molecular Biology, College of Marine Life Sciences, Ocean University of China, Qingdao, People’s Republic of China; c Department of Microbiology, Faculty of Veterinary Medicine, Zagazig University, Zagazig, Egypt; d Department of Biomedical and Diagnostic Sciences, University of Tennessee, Knoxville, Tennessee, USA; e College of Food Science and Engineering, Ocean University of China, Qingdao, China; f Processes Design and Development Department, Egyptian Petroleum Research Institute, Nasr City, Cairo, Egypt; g Department of Basic Medical Sciences, Shaqra University, Shaqraa, Kingdom of Saudi Arabia; h Agriculture Research Center, Animal Health Research, Zagazig, Egypt; i Department of Clinical Laboratory Sciences, College of Applied Medical Sciences, Taif University, Taif, Saudi Arabia; j General Practitioner, Faculty of Medicine, University of Gezira, Wad Medani, Sudan; k Department of Medical Laboratory Techniques, Al Maarif University College, Al Anbar, Ramadi, Iraq; l Department of Clinical Laboratory Sciences, College of Applied Medical Sciences, Jouf University, Sakaka, Saudi Arabia; m Department of Biology, College of Science, Princess Nourah Bint Abdulrahman University, Riyadh, Saudi Arabia; n Botany and Microbiology Department, Faculty of Science, Beni-Suef University, Beni-Suef, Egypt; o Department of Biotechnology, Faculty of Science and Technology, Shendi University, Shendi, Nher Anile, Sudan; p Department of Medical Microbiology and Immunology, Faculty of Medicine, Zagazig University, Zagazig, Egypt; University of Calgary

**Keywords:** rhamnolipids, biofilm formation, antiadhesive property, iron oxide nanoparticles, drug delivery, antimicrobial activity

## Abstract

Surface-growing antibiotic-resistant pathogenic bacteria such as Escherichia coli and Staphylococcus aureus are emerging as a global health challenge due to dilemmas in clinical treatment. Furthermore, their pathogenesis, including increasingly serious antimicrobial resistance and biofilm formation, makes them challenging to treat by conventional therapy. Therefore, the development of novel antivirulence strategies will undoubtedly provide a path forward in combatting these resistant bacterial infections. In this regard, we developed novel biosurfactant-coated nanoparticles to combine the antiadhesive/antibiofilm properties of rhamnolipid (RHL)-coated Fe_3_O_4_ nanoparticles (NPs) with each of the *p*-coumaric acid (*p*-CoA) and gallic acid (GA) antimicrobial drugs by using the most available polymer common coatings (PVA) to expand the range of effective antibacterial drugs, as well as a mechanism for their synergistic effect via a simple method of preparation. Mechanistically, the average size of bare Fe_3_O_4_ NPs was ~15 nm, while RHL-coated Fe_3_O_4_@PVA@*p*-CoA/GA was about ~254 nm, with a drop in zeta potential from −18.7 mV to −34.3 mV, which helped increase stability. Our data show that RHL-Fe_3_O_4_@PVA@*p*-CoA/GA biosurfactant NPs can remarkably interfere with bacterial growth and significantly inhibited biofilm formation to more than 50% via downregulating *IcaABCD* and *CsgBAC* operons, which are responsible for slime layer formation and curli fimbriae production in S. aureus and E. coli, respectively. The novelty regarding the activity of RHL-Fe_3_O_4_@PVA@*p*-CoA/GA biosurfactant NPs reveals their potential effect as an alternative multitarget antivirulence candidate to minimize infection severity by inhibiting biofilm development. Therefore, they could be used in antibacterial coatings and wound dressings in the future.

**IMPORTANCE** Antimicrobial resistance poses a great threat and challenge to humanity. Therefore, the search for alternative ways to target and eliminate microbes from plant, animal, and marine microorganisms is one of the world’s concerns today. Furthermore, the extraordinary capacity of S. aureus and E. coli to resist standard antibacterial drugs is the dilemma of all currently used remedies. Methicillin-resistant S. aureus (MRSA) and vancomycin-resistant S. aureus (VRSA) have become widespread, leading to no remedies being able to treat these threatening pathogens. The most widely recognized serotypes that cause severe foodborne illness are E. coli O157:H7, O26:H11, and O78:H10, and they display increasing antimicrobial resistance rates. Therefore, there is an urgent need for an effective therapy that has dual action to inhibit biofilm formation and decrease bacterial growth. In this study, the synthesized RHL-Fe_3_O_4_@PVA@*p*-CoA/GA biosurfactant NPs have interesting properties, making them excellent candidates for targeted drug delivery by inhibiting bacterial growth and downregulating biofilm-associated *IcaABCD* and *CsgBAC* gene loci.

## INTRODUCTION

Antimicrobial resistance is limiting treatment choices for Staphylococcus aureus, one of the most common Gram-positive bacteria linked to a number of life-threatening local and systemic illnesses (1). Methicillin-resistant S. aureus (MRSA) is a result of the bacteria’s extraordinary capacity to resist standard antibacterial drugs (2). Currently, vancomycin is used to deal with MRSA-associated infection (3). However, therapy options by vancomycin are severely constrained, and vancomycin-resistant S. aureus (VRSA) has been substantially responsible for significant clinical problems, and accordingly, no remedies will be reachable for MRSA or VRSA in the future (4).

Escherichia coli is Gram-negative bacteria accountable for an extensive range of community-acquired extraintestinal infections, and it displays increasing antimicrobial resistance rates (5); E. coli O157:H7, O26:H11, and O78:H10 are serotypes that cause severe foodborne illness (6, 7). Developing a new therapeutic strategy to replace traditional antibiotic usage regimens in the treatment of drug-resistant bacterial infections is a potential approach (8).

Microbial biofilms are surface-attached microbial colonies on a cell that contribute to bacterial persistence on different surfaces, protect the microbes from adverse environmental conditions, and result in infection, accordingly slowing wound healing and making it more difficult to treat infections (9, 10). E. coli biofilm consists of thin, flexible aggregative protein filaments known as curli fibrils that allow binding to several extracellular polymeric substances (EPSs) (11) and are encoded by *CsgBAC* operons, including *crl*, *csgA*, and *csgD* (12). On the other hand, poly-*N*-acetyl-(1-6)-β-glucosamine (PNAG) has been established as a vital constituent of S. aureus biofilms that are encoded by the intercellular adhesion (*IcaABCD*) gene locus (13).

Biofilm formation can be inhibited by self-cleaning and drug-tethered surfaces (14, 15). Surface functionalization, such as impregnation, and coating with nanomaterials have numerous applications in biomedical fields due to their potential as an inhibitory tool, including antibacterial and antibiofilm properties (16).

*p*-Coumaric acid (*p*-CoA) is a hydroxy derivative of cinnamic acid that represents an important group of phenolic compounds (17). It has versatile biological activity, including antioxidant, anticancer, antimicrobial, antiviral, anti-inflammatory, and antiplatelet properties. (18–21).

Gallic acid (GA) (3,4,5-trihydroxybenzoic acid) is a well-known vigorous natural antioxidant compound found abundantly in various herbs (22). It possesses unique physicochemical characteristics, such as nontoxicity, biodegradability, and availability; therefore, it is used as a multitherapeutic agent, with antioxidant, anti-inflammatory, anticarcinogenic, and antimicrobial properties (23).

Rhamnolipids (RHLs) are low-molecular-weight amphiphilic anionic glycolipid biosurfactants with one or two rhamnose moieties in the hydrophilic head and one or two fatty acid chains in the hydrophobic tail in monorhamnolipids (Rha-C_10_-C_10_) or dirhamnolipids (Rha-Rha-C_10_-C_10_), respectively (24–27).

RHLs are of special importance as green biological agents produced from bacteria, and they have biocompatibility and nontoxicity properties (28, 29). It has been stated that RHLs have antibiofilm and antimicrobial properties that interact with a variety of bacteria, including Gram-negative bacteria (Helicobacter pylori, E. coli, and Salmonella), Gram-positive bacteria (Staphylococcus aureus, Bacillus pumilus, and Listeria monocytogenes), and fungal strains (Yarrowia lipolytica) (30, 31).

In the era of nanotechnology, metals and metal oxide nanoparticles (NPs) (mainly Fe_3_O_4_) have generated extreme interest in biomedical applications due to their unique physical and chemical properties such as nanometer size, great bioavailability, and ability to interact on cell surfaces and cross cell membranes (22, 32, 33). Multiple mechanisms of the antibacterial action of nanoparticles have been proposed; smaller particle size and greater surface area of nanoparticles lead to its better and strongly adhere to the bacterial cell and then release metal ions that induce oxidative stress by free radical formation (34–36), causing loss of membrane integrity, disruption, and eventually killing the bacteria (32, 37).

Several studies have proven that the attachment of antioxidants to nanoparticle surfaces increases antioxidant activity and bioavailability for long periods (22). In this way, several methodologies have been advanced to coat Fe_3_O_4_ during the preparation (endo situ) and next to synthesis with polyvinyl alcohol (PVA) as the most common coatings (38).

**FIG 1:**
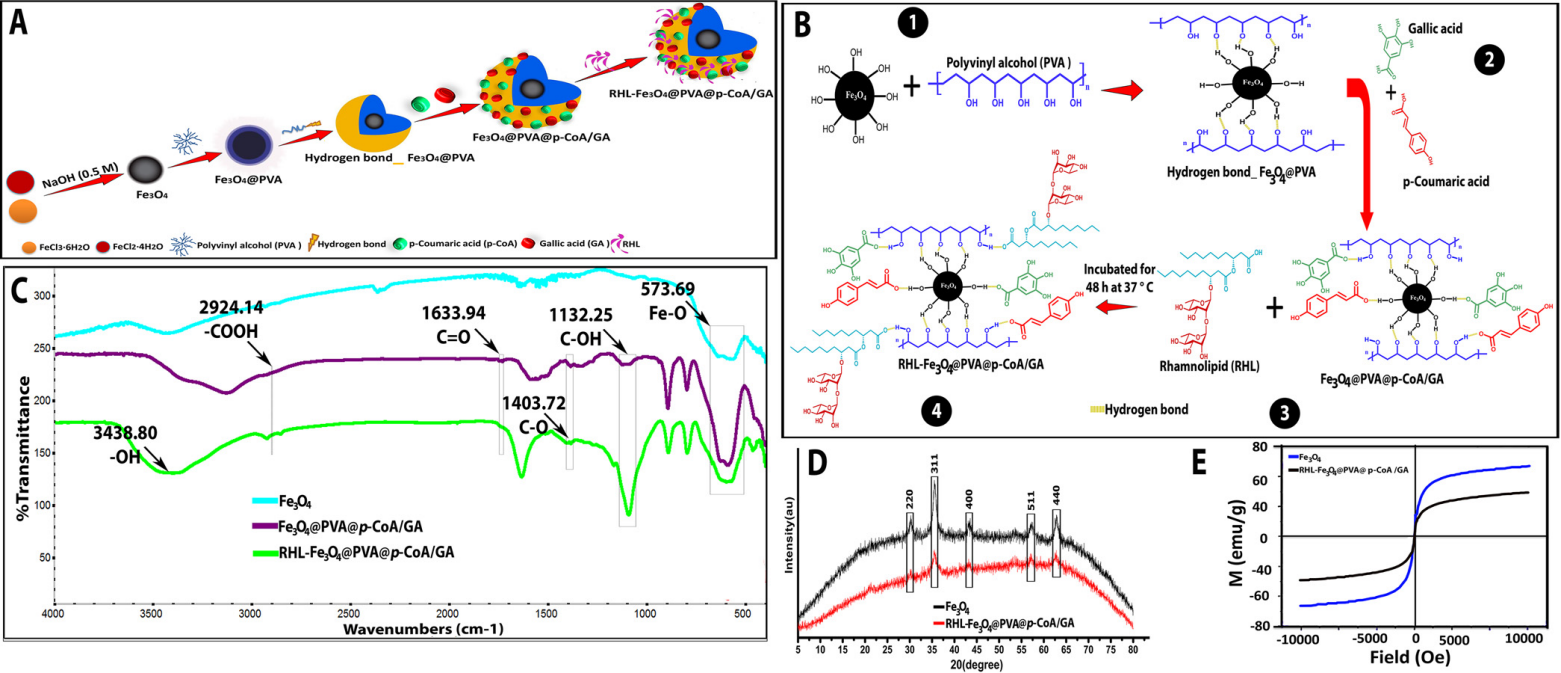
(A) Schematic illustration of prepared RHL-Fe_3_O_4_@PVA@*p*-CoA/GA biosurfactant magnetic NPs according to the emulsion-coacervation method at 20 to 22°C. (B) Exchange of chemosorbed rhamnolipid (RHL) ligands on the Fe_3_O_4_@PVA@*p*-CoA/GA NP surfaces via hydrogen binding donor. (C to E) FTIR spectra (C), XRD pattern (D), and VSM analysis (E) of bare Fe_3_O_4_ and Fe_3_O_4_@PVA@*p*-CoA/GA NPs and RHL-Fe_3_O_4_@PVA@*p*-CoA/GA biosurfactant NPs.

In this study, as shown in Fig. 1A, we hypothesized that creating an RHL core shell of Fe_3_O_4_ NP surfaces and loading with *p*-CoA and GA natural antimicrobials by using the most available polyvinyl alcohol (PVA) would greatly improve their antiadhesive, antibiofilm, and antimicrobial properties, using Gram-negative bacteria (E. coli) and Gram-positive bacteria (S. aureus) as model microorganisms, where the iron atoms coordinate with H_2_O, which readily dissociates to configure Fe-OH and leaves, on the Fe_3_O_4_ NP surfaces, hydroxyl functionalized groups. Followed by coating with a solution composed of a PVA polymer, it can be stelled by physical intermolecular or covalent cross-linking, which, in most cases, can be carried out by changing the pH to 7.3 and lowering temperature. Furthermore, the presence of hydroxyl groups on the surfaces of magnetic nanoparticles provides a flexible silicone handle allowing attachment with PVA, and it reacts to the effective groups to both GA and *p*-CoA by forming hydrogen binding donors (Fig. 1B). Furthermore, the transcription of *csgBAC* operons, which encode the synthesis of curli fimbriae, and the *ica* operon, which encodes the synthesis of polysaccharide intercellular adhesion as an important component for biofilm formation (12, 13), revealed the importance of studying the transcriptional modulatory effectiveness on biofilm-associated genes, polysaccharide production (*icaA* and *icaD*), and curli structural subunits (*csgA*, *csgD*, and their regulator, *crl*) for S. aureus and E. coli, respectively.

## RESULTS

### Characterization of RHL-Fe_3_O_4_@PVA@*p*-CoA/GA biosurfactant NPs.

Here, the characteristic Fourier transform infrared spectroscopy (FTIR) absorption peaks at 574 to 610 cm^−1^ in Fe_3_O_4_ are consistent with the asymmetric and symmetric stretching vibration of Fe-O. Infrared (IR) spectra for pure GA and pure *p*-CoA showed a band at 1,634 cm^−1^ for C=O stretching of the COOH group and broad bands in the range of 2,800 to 3,500 cm^−1^ for both carboxylic and phenolic −OH groups. Furthermore, IR spectra for RHL-Fe_3_O_4_@PVA@*p*-CoA/GA biosurfactant NPs are show a C-O band of 1,402 cm^−1^, along with the broad phenolic −OH band. This indicates the formation of a covalent C-O bond and confirms the formation of a GA and *p*-CoA coat onto the Fe_3_O_4_ surface (Fig. 1C). The X-ray diffraction patterns for the bare iron oxide nanoparticles and iron oxide nanoparticles coated with GA and *p*-CoA are shown in Fig. 1D. For both samples Fe_3_O_4_ and RHL-Fe_3_O_4_@PVA@p-CoA/GA NPs, six characteristic peaks observed at 20° 30.3°, 35.8°, 43.5°, 53.7°, 57.4°, and 62.9° can be specified as 220, 311, 400, 422, 511, and 440, respectively. To measure the magnetic property of Fe_3_O_4_ and RHL-Fe_3_O_4_@PVA@*p*-CoA/GA NPs, magnetization as a function of the applied magnetic fields is demonstrated in Fig. 1E, which indicates that both are superparamagnetic. However, Fe_3_O_4_ NPs presented a saturation magnetization of 68.9 electromagnetic units (emu) per gram, and RHL-Fe_3_O_4_@PVA@*p*-CoA/GA NPs had a saturation of 49.4 emu per gram. However, RHL-Fe_3_O_4_@PVA@*p*-CoA/GA biosurfactant NPs showed a decrease in saturation magnetization and could still be efficiently detached from the medium.

### RHL-Fe_3_O_4_@PVA@*p*-CoA/GA nanoparticle competition dispersions.

**FIG 2:**
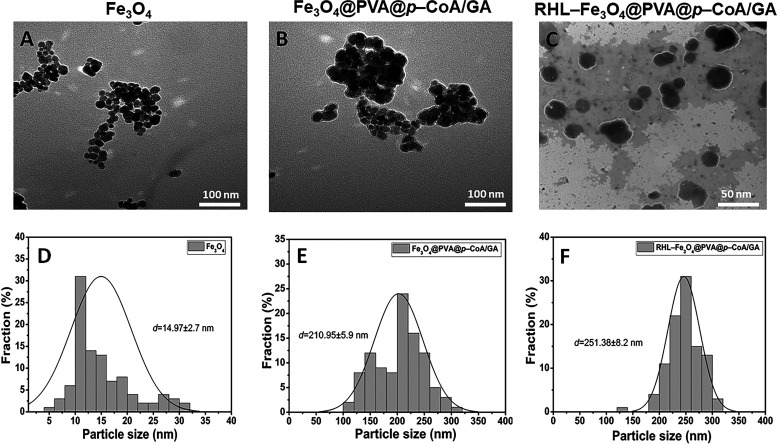
Average particle size and size distribution of prepared samples. (A and D) Bare Fe_3_O_4_ (scale bar, 100 nm); (B and E) Fe_3_O_4_@PVA@*p*-CoA/GA NPs (scale bar, 100 nm); (C and F) RHL-Fe_3_O_4_@PVA@*p*-CoA/GA NPs (scale bar, 50 nm) measured by TEM. Data of size distribution are presented as means ± SD (*n *=* *3).

Dynamic light scattering (DLS) was used to measure particle size (PS), polydispersity index (PDI), and zeta potential (ZP) of bare Fe_3_O_4_, RHL-Fe_3_O_4_, Fe_3_O_4_@PVA@*p*-CoA/GA, and RHL-Fe_3_O_4_@PVA@*p*-CoA/GA NPs. The mean values recorded for all the systems showed a PS distribution in nanometers as shown in Fig. 2 and Table S1 in the supplemental material. The findings show Fe_3_O_4_ nanoparticles have sizes around ~15.09 nm with a positive potential of −18.7 mV, while Fe_3_O_4_@PVA nanoparticles are larger, around 25 nm, and have a zeta potential of −33.3 mV. When the *p-*CoA and GA nanoparticles are loaded onto Fe_3_O_4_@PVA, the PS increases to ~213.8 nm, and the ZP becomes −33.7 mV. When the Fe_3_O_4_@PVA@*p*-CoA/GA NPs are simultaneously loaded with RHL (RHL-Fe_3_O_4_@PVA@*p*-CoA/GA NPs), the PS becomes ~254.6 nm, and the ZP becomes −34.3 mV. Additionally, the loading eﬃciencies of *p-*CoA and GA in the RHL-Fe_3_O_4_@PVA NPs are 95 ± 2.8% and 97 ± 2.2%, respectively. On the other hand, the determined PDI values were 0.385, 0.153, 0.264, and 0.202 for Fe_3_O_4_, Fe_3_O_4_@PVA, Fe_3_O_4_@PVA@*p*-CoA/GA, and RHL-Fe_3_O_4_@PVA@*p*-CoA/GA NPs, respectively.

**FIG 3:**
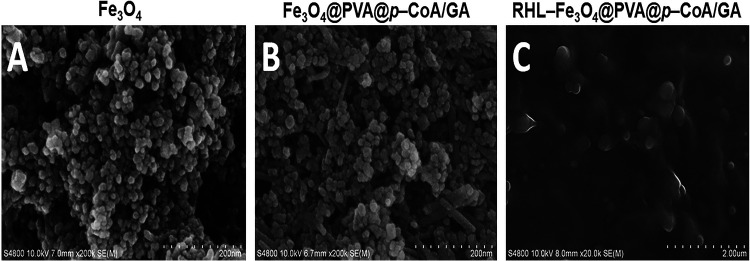
SEM analysis of prepared NPs. (A to C) Bare Fe_3_O_4_ (A), Fe3O4@PVA@*p*-CoA/GA (B), and RHL-Fe_3_O_4_@PVA@*p*-CoA/GA (C) NPs (scale bar, 200 nm).

The morphological surface was observed by transmission electron microscopy (TEM), as shown in Fig. 2, and scanning electron microscopy (SEM), as shown in Fig. 3. Bare Fe_3_O_4_ showed the particles have a spherical shape and uniform size distribution with an aggregation of TEM (Fig. 2A and D) and SEM (Fig. 3A) images, while the Fe_3_O_4_@PVA@*p*-CoA/GA NP images demonstrated a core shell structure with a black core surrounded by a gray layer of TEM (Fig. 2B and E) and SEM (Fig. 3B) images. Furthermore, RHL-Fe_3_O_4_@PVA@*p*-CoA/GA NP images showed spherical, well-dispersed nonagglomeration with a white layer around the darker-loaded Fe_3_O_4_@PVA polymer due to the presence of a lipid layer of RHL-encapsulated Fe_3_O_4_@PVA@*p*-CoA/GA NPs; these results confirmed the success of the capsulation process of TEM (Fig. 2C and F) and SEM (Fig. 3C) images.

### *In vitro* drug release.

**FIG 4:**
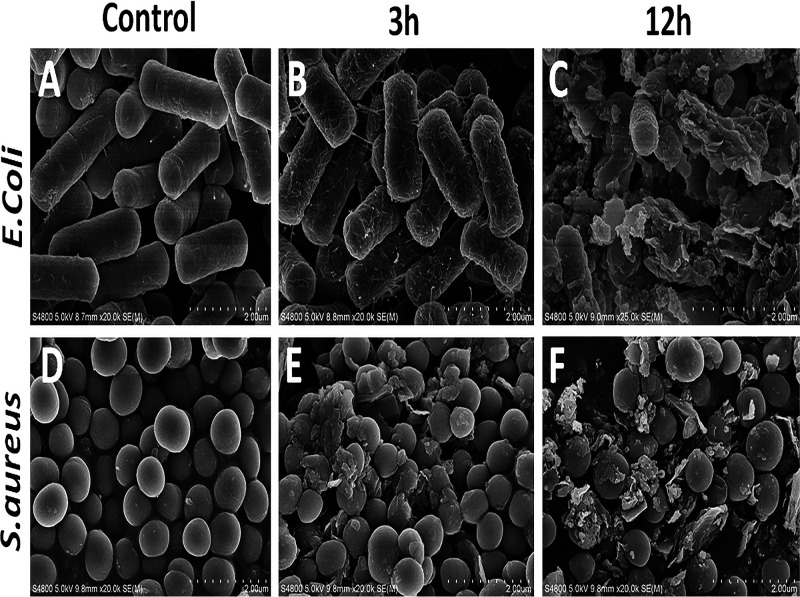
*In vitro* drug release evaluation shown as the percentage of cumulative release and TEM. (A) Release profile of both *p*-CoA and GA for prepared RHL-Fe_3_O_4_@PVA@*p*-CoA/GA NPs at pH 1.2, 6.8, and 7.4 at 37°C. (B) TEM images of the change in the shape and size of RHL-Fe_3_O_4_@PVA@*p*-CoA/GA NPs with release of the drugs at pH values of 1.2, 6.8, and 7.4 after 3, 5, and 48 h, respectively, at 37°C (scale bar, 2 μm).

Here, *p*-CoA and GA were encapsulated in the prepared RHL-Fe_3_O_4_@PVA NPs to prevent their degradation in gastric acid and to induce their release in or under the mucus layer; the results are shown in Fig. 4. The drug release test was performed at different pH levels of phosphate-buffered saline (PBS) solutions at 37°C to simulate the pH conditions, such as pH 1.2 for gastric acid, pH 6.8 for the mucus layer, and pH 7.4 for the gastric epithelium environment. At pH 1.2, only ~37% and ~40% of the *p-*CoA and GA are released from the RHL-Fe3O4@PVA@*p*-CoA/GA biosurfactant NPs after 3 h incubation, while the *p-*CoA and GA released had increased to ~35% and ~48% at pH 6.8, respectively, after 5 h incubation. Furthermore, a sustained and controlled releasing profile is accomplished at pH 7.4, and the release of *p-*CoA and GA increased to ~78% and ~80% after 24 h incubation, respectively, and ~96% and ~96% after 48 h incubation, respectively (Fig. 4A), which was predicted by TEM images that show increased and distorted RHL-Fe_3_O_4_@PVA@*p*-CoA/GA NP particle size at different simulated pH conditions and 37°C (Fig. 4B).

### Bactericidal effect of RHL-Fe_3_O_4_@PVA@*p*-CoA/GA biosurfactant NPs on S. aureus and E. coli.

**FIG 5:**
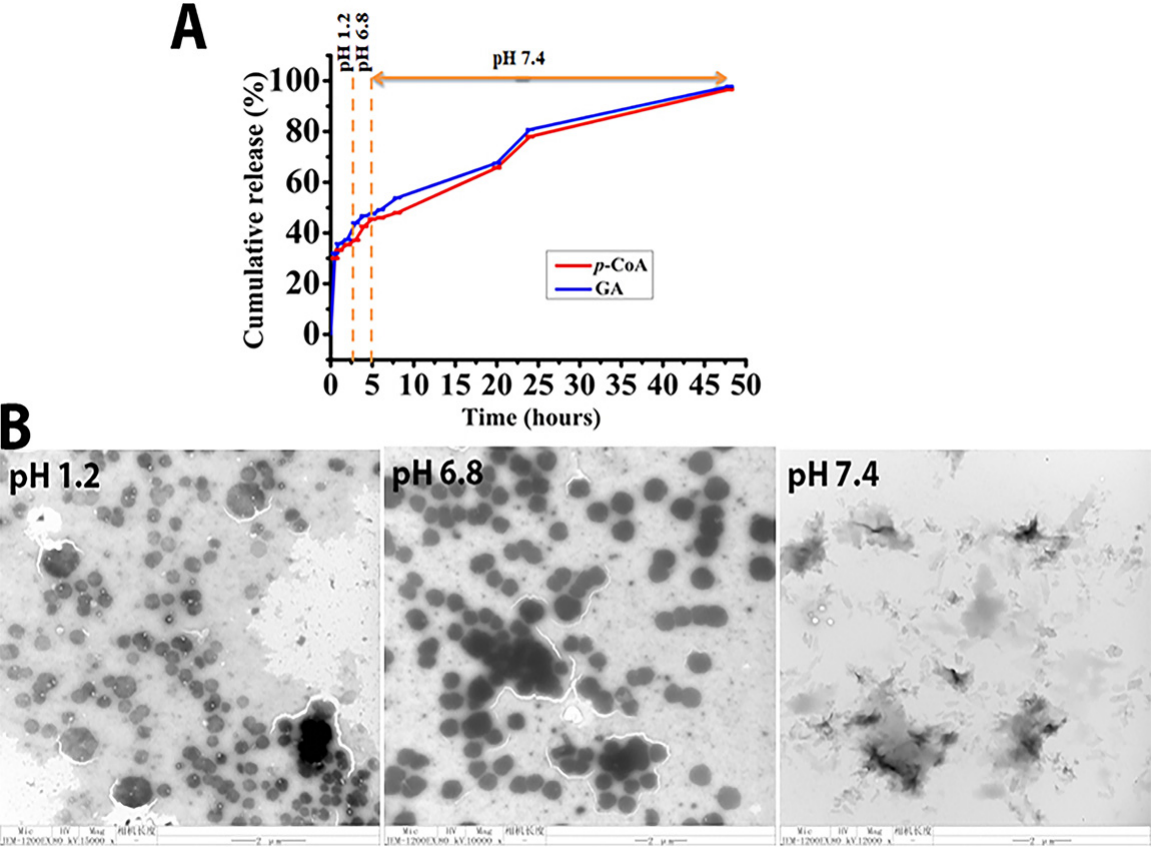
RHL-Fe_3_O_4_ and RHL-Fe_3_O_4_@PVA@p-CoA/GA NPs inhibit E. coli and S. aureus bacterial growth. Inhibitory effects of RHL-Fe_3_O_4_ and RHL-Fe_3_O_4_@PVA@p-CoA/GA NPs compared with imipenem and vancomycin as standard antibiotic control were determined *in vitro* by agar well diffusion assay. (A) E. coli O157:H7, O26:H11, and O78:H10; (B) MSSA, MRSA, and VRSA. Each column shows the mean ± SD of three independent experiments, and representative images are shown. Asterisk represents statistically significant differences (*P* < 0.05), and “ns” represents nonsignificant differences (*P* > 0.05) compared to the control sample.

Antibacterial activity of RHL-Fe_3_O_4_@PVA@*p*-CoA/GA biosurfactant NPs compared to the standard antibiotics against the selected S. aureus and E. coli, which represented Gram-positive and Gram-negative bacteria, respectively, was assessed by evaluating the diameter of inhibition zones (millimeters) and MICs (39) (Fig. 5 and Table 1). The standard antimicrobial agents used as conventional drugs are frequently used as the last drug of choice; all E. coli serotypes were susceptible to imipenem, while all S. aureus isolates were susceptible to vancomycin except VRSA. As a negative control, no inhibition zones were developed with all microbial strains with D.W. as diluent of biosurfactant NPs. RHL-Fe_3_O_4_@PVA@*p*-CoA/GA biosurfactant NPs exhibited a significant inhibitory effect against all of the tested strains, with inhibition zone diameters up to 45 mm (Fig. 5). The maximum antibacterial activity was observed against methicillin-susceptible S. aureus (MSSA) and E. coli O26:H11, with inhibition zone diameters of 40 mm and 45 mm compared with 30 mm and 25 mm inhibition zone diameters of vancomycin and imipenem antibiotic control, respectively. Lower antibacterial activity was depicted against VRSA and E. coli O157:H7, with diameters of 25 mm and 35 mm, respectively (Fig. 5). As expected, RHL-Fe_3_O_4_ without PVA@*p*-CoA/GA biosurfactants had a lower inhibitory effect on the tested microorganisms (Fig. 5).

**TABLE 1:** Sub-MICs, MICs, and MBCs of biosurfactant magnetic nanoparticles against S. aureus and E. coli isolates

Isolate	Data (μg/mL^−1^) for:								
	Control^*a*^			RHL-Fe_3_O_4_ (μg/mL^−1^)			RHL-Fe_3_O_4_@PVA@*p*-CoA/GA (μg/mL^−1^)		
	Sub-MIC	MIC	MBC	Sub-MIC	MIC	MBC	Sub-MIC	MIC	MBC
E. coli strains									
O157:H7	4	8	16	32	64	128	16	32	64
O26:H11	0.25	0.5	1	2	4	8	0.5	1	2
O78:H10	2	4	8	16	32	64	4	16	32
S. aureus strains									
MSSA	0.125	0.25	0.5	4	16	32	2	4	8
MRSA	0.5	1	2	16	32	64	4	16	32
VRSA	32	64	128	32	64	128	16	32	64

a Imipenem is the control for E. coli, and vancomycin is the control for S. aureus.

This efficacy was confirmed with MICs of RHL-Fe_3_O_4_ ranging from 4, 32, and 64 μg mL^−1^ for E. coli O26:H11, O78:H10, and O157:H7, respectively (Table 1), while the MICs were 16, 32, and 64 μg mL^−1^ for MSSA, MRSA, and VRSA, respectively. MIC values decreased when RHL-Fe_3_O_4_was loaded on PVA@*p*-CoA/GA biosurfactant NPs to 32 μg mL^−1^ for E. coli O157:H7 and VRSA, and those of E. coli O78:H10 and MRSA decreased to 16 μg mL^−1^ (Table 1). The lowest MICs of 1 to 4 μg mL^−1^ were recorded for E. coli O26:H11 and MSSA, respectively. Furthermore, minimum bactericidal concentration (MBC) values of all screened isolates for unloaded and loaded biosurfactant NPs were 2-fold higher than their corresponding MIC values, indicating their bactericidal effect (Table 1).

### Scanning electron microscopy analysis.

**FIG 6:**
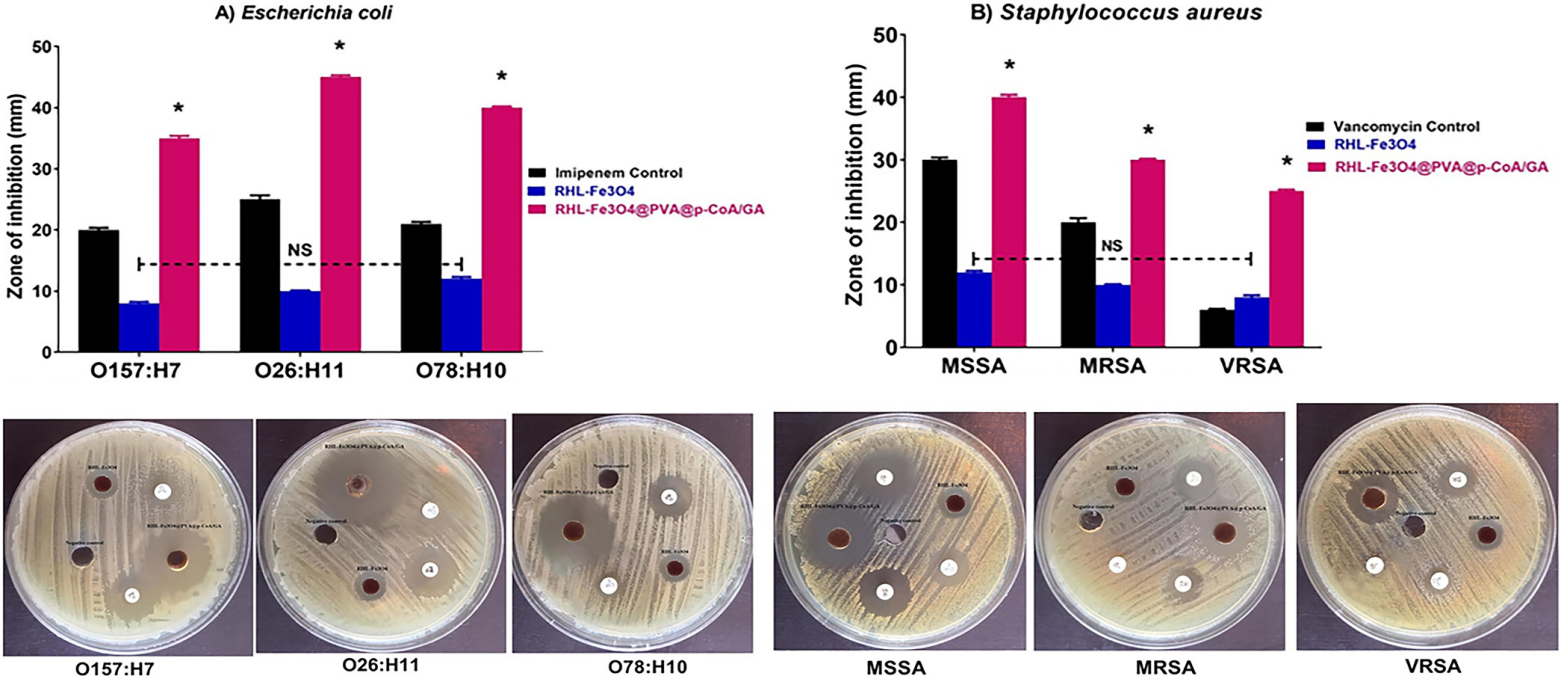
SEM images of E. coli and S. aureus after 3 and 12 h exposure to the different treatments. (A and D) Untreated (control); (B and E) treatment with MICs of RHL-Fe_3_O_4_@PVA@*p*-CoA/GA biosurfactant NPs after 3 h; (C and F) treatment with MICs of RHL-Fe_3_O_4_@PVA@*p*-CoA/GA biosurfactant NPs after12 h (scale bar, 2 μm).

SEM images indicated that the presence of RHL-Fe_3_O_4_@PVA@*p*-CoA/GA biosurfactant NPs caused massive morphological changes and bactericidal effects against E. coli O78:H10 and MRSA (Fig. 6). Furthermore, this effect is time dependent, as illustrated by enormous deterioration, seriously damaged cell wall, rupture, and bacterial death at 12 h compared with only slight surface cracks due to a broken external wall at 3 h. In contrast, microscopy evaluations confirm that E. coli and S. aureus cells without treatment increased and aggregated with the cell membranes smooth and intact (Fig. 6).

### Transmission electron microscopy analysis.

**FIG 7:**
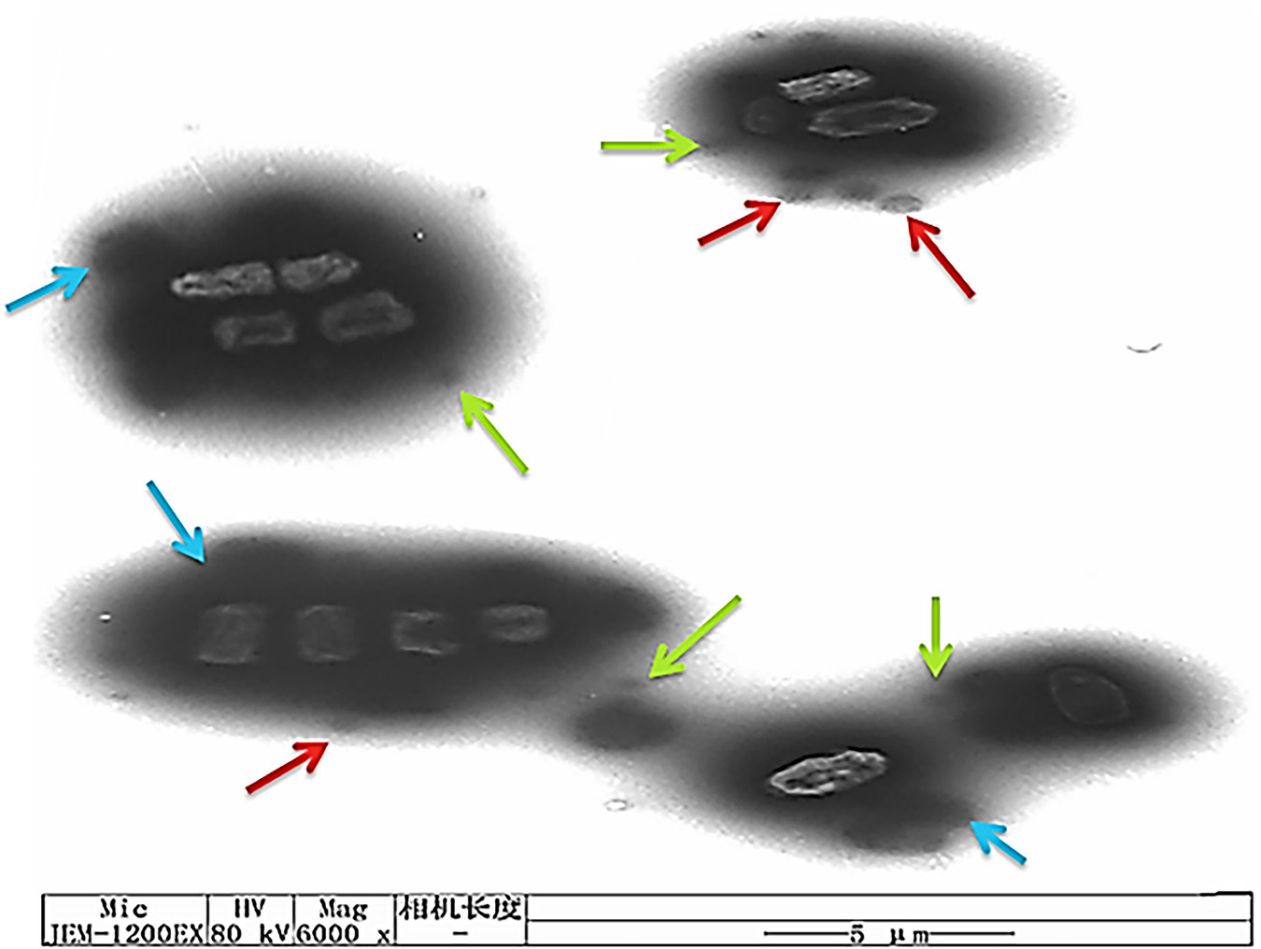
Mechanism of action between RHL-Fe_3_O_4_@PVA@*p*-CoA/GA biosurfactant NPs and E. coli bacteria cell membrane, measured by TEM (scale bar, 5 μm).

TEM was used to further investigate the targeting mechanism between RHL-Fe_3_O_4_@PVA@*p*-CoA/GA with the E. coli outer membrane, confirming that their action affecting cell morphology is related to cell death (Fig. 7). TEM results showed the targeting and elimination of E. coli via the remarkable closeness of RHL-Fe_3_O_4_@PVA@*p*-CoA/GA biosurfactant NPs and their adhesion to the outer membrane due to the surface potential of RHL-coated NPs (as indicated by red and green outlined arrows in Fig. 7), which leads to damage and punctuate cells through leakage of cytoplasmic and nuclear materials (as indicated by blue outlined arrows in Fig. 7).

### Initial adhesion, biofilm development, and transcriptional profiles in the presence of RHL-Fe_3_O_4_@PVA@*p*-CoA/GA biosurfactant NPs.

**FIG 8:**
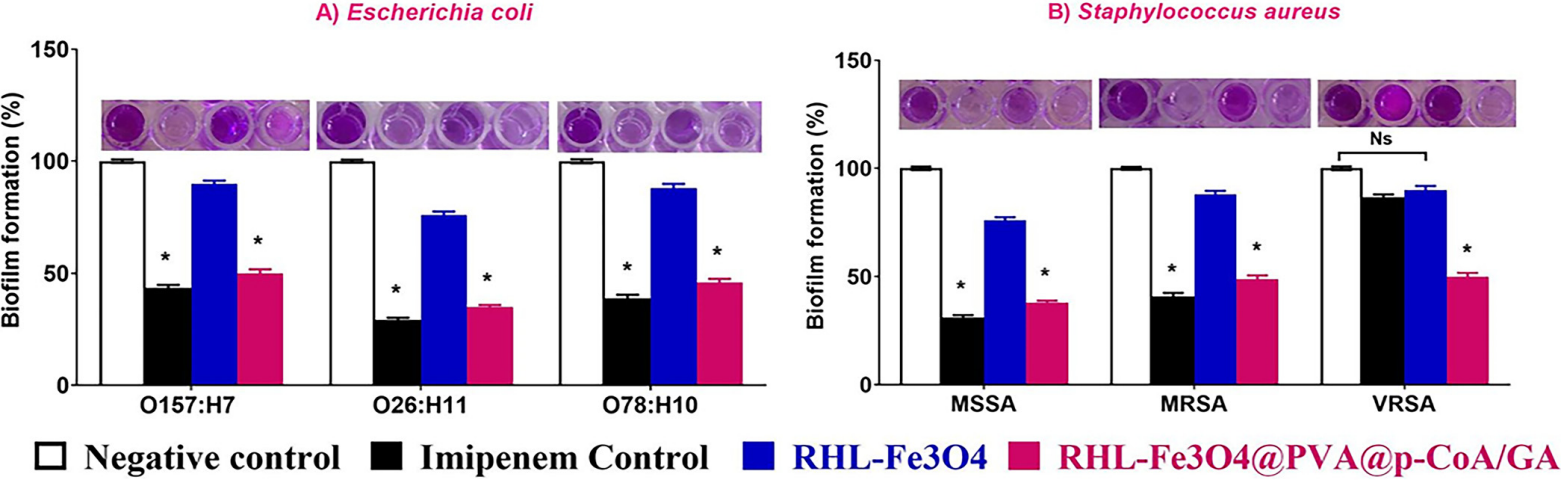
RHL-Fe_3_O_4_ and RHL-Fe_3_O_4_@PVA@*p*-CoA/GA NPs reduce the initial adhesion and biofilm formation of E. coli O157:H7, O26:H11, and O78:H10 (A) and S. aureus MSSA, MRSA, and VRSA (B). The biofilms of treated bacteria were detected by crystal violet staining and quantified by measuring the OD_600_. Each column shows the mean ± SD of three independent experiments, and representative images are shown; increasing violet color indicates higher biofilm formation. Asterisk represents statistically significant differences (*P* < 0.05), and “ns” represents nonsignificant differences (*P* > 0.05) compared to the control sample.

**FIG 9:**
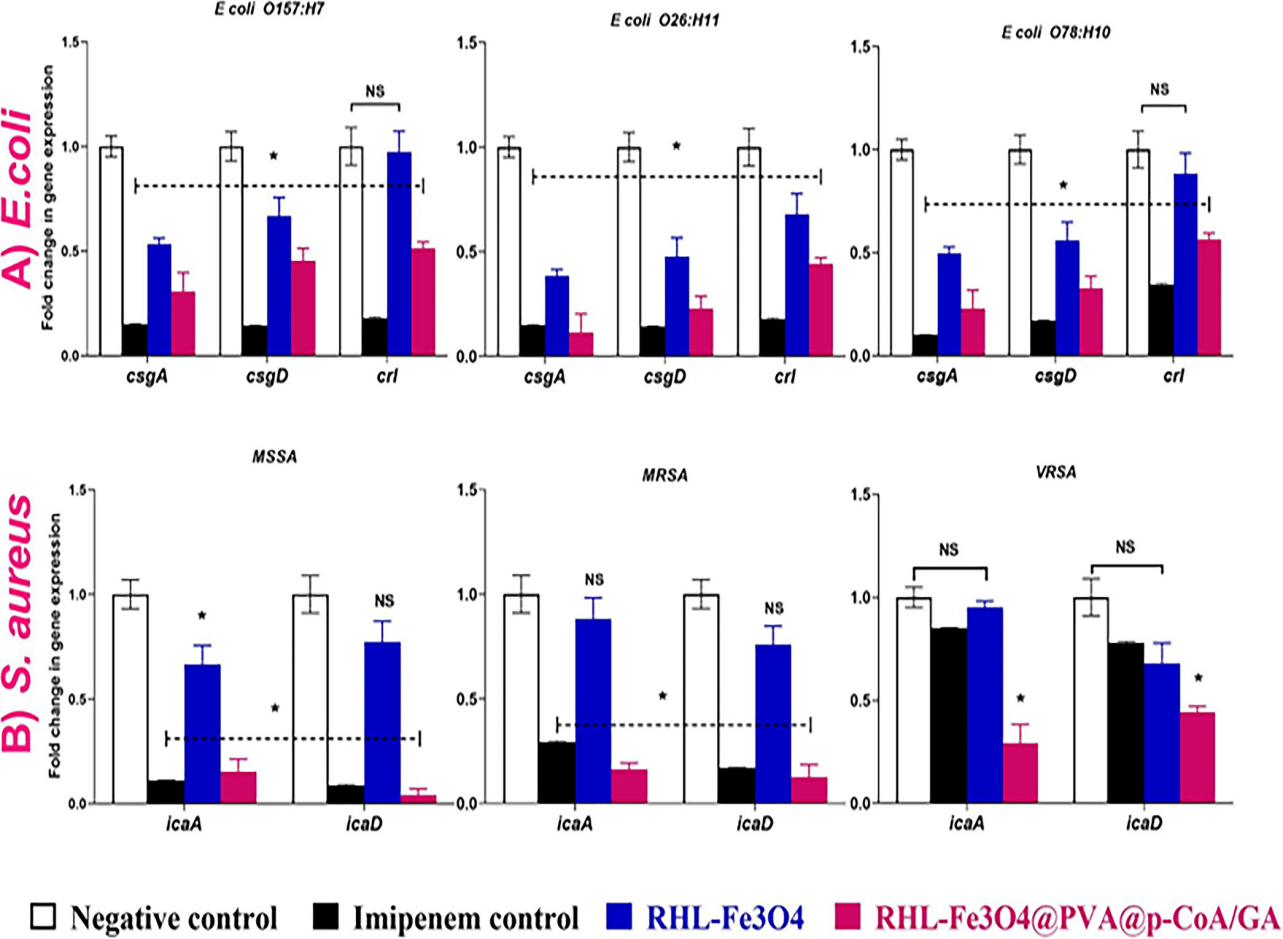
Transcriptional profile of biofilm-associated genes upon treatment with RHL-Fe_3_O_4_@PVA@*p*–CoA/GA NPs. (A) E. coli O157:H7, O26:H11, and O78:H10; (B) MSSA, MRSA, and VRSA. Relative gene expression levels of *csgA*, *csgD*, *crl*, *icaA*, and *icaD* were calculated using the ΔΔ*CT* method and expressed as fold change. 16S rRNA was used as the endogenous control. Each column shows the mean ± SD of three independent experiments Asterisk represents statistically significant differences (*P* < 0.05), and “ns” represents nonsignificant differences (*P* > 0.05) compared to the control sample.

To investigate whether the biosurfactant NPs hinder biofilm formation in S. aureus and E. coli, we evaluated sub-MIC concentrations of the two compounds along with the control antibiotics (Fig. 8). Our results revealed good antibiofilm activity (compared with untreated negative control that showed 100% biofilm formation) in which RHL-Fe_3_O_4_@PVA@*p*-CoA/GA significantly inhibited S. aureus and E. coli biofilm formation by more than 50% (35 to 38% for E. coli O26:H11 and MSSA, respectively), and those of E. coli O157:H7 and VRSA decreased to 50%. However, the same inhibition profile was not observed when biofilms were exposed to unloaded RHL-Fe_3_O_4_. In fact, RHL-Fe_3_O_4_ only showed a minor effect on S. aureus and E. coli bacterial growth (Fig. 8) and also was not able to significantly avoid initial bacterial adhesion (Fig. 8). Furthermore, to investigate the inhibitory effect of RHL-Fe_3_O_4_@PVA@*p*-CoA/GA biosurfactant NPs, real-time reverse transcription-quantitative PCR (qRT-PCR) was used to determine the modulatory effect on biofilm-associated genes, including polysaccharide production (*icaA* and *icaD*) and curli structural subunits (*csgA*, *csgD*, and their regulator, *crl*) for S. aureus and E. coli, respectively (Fig. 9). While unloaded RHL-Fe_3_O_4_ treatment of all E. coli serotypes resulted in 0.3- to 0.9-fold change for *csgA*, *csgD*, and *crl* genes, respectively, RHL-Fe_3_O_4_@PVA@*p*-CoA/GA biosurfactant NPs caused significant downregulation of *csgA*, *csgD*, and *crl* genes (up to 0.1 to 0.5; *P* < 0.05) compared to untreated biofilm-producing isolate (Fig. 9A). Moreover, the highest modulation and reduction in biofilm-associated *csgA*, *csgD*, and *crl* genes were observed in RHL-Fe_3_O_4_@PVA@*p*-CoA/GA-treated E. coli O26:H11, with 0.1-, 0.2-, and 0.4-fold changes, respectively (Fig. 9A). We observed only minor and not significant effects of S. aureus treated with unloaded RHL-Fe_3_O_4_ on *icaA* and *icaD* genes responsible for polysaccharide production (up to 0.67 to 0.95; *P *>* *0.05) (Fig. 9B). The significantly higher efficacy of RHL-Fe_3_O_4_@PVA@*p*-CoA/GA treatment over vancomycin antibiotic control in VRSA was detected by downregulation of *icaA* and *icaD* genes (0.2 to 0.4; *P *<* *0.05) (Fig. 9B).

## DISCUSSION

Antibiotic-resistant microorganisms have become a serious threat to human health (40). The continuous emergence of methicillin-resistant S. aureus (MRSA) and vancomycin-resistant S. aureus (VRSA) makes it challenging to be treated by conventional therapy (4). Simultaneously, Escherichia coli serotypes O157:H7, O26:H11, and O78:H10 are the biggest cause of severe foodborne illness that are displaying increasing antimicrobial resistance rates (6). Moreover, biofilms are an exact mechanism of bacterial persistence and antibacterial resistance, as antimicrobial drugs in solution only penetrate and kill the bacteria outside the biofilm (41). In this sense, the development of nanoparticles is gaining traction, and they are excellent tools for overcoming the therapeutic difficulty accompanied by multidrug resistance and biofilm persistence of bacteria. As reported in several studies, the nanoparticles exhibit broad-spectrum antimicrobial activity for both Gram-positive and Gram-negative bacteria (42–44). The purpose of this study was to design a novel RHL core shell of Fe_3_O_4_ NPs, loaded with *p*-CoA and GA, by using a PVA-coated polymer, and evaluate their activity as an alternative multitarget candidate to inhibit S. aureus and E. coli growth and biofilm formation.

An FTIR result showed confirmation of successfully loading GA and *p-*CoA onto the surface of magnetic nanoparticles (45, 46). Moreover, X-ray diffraction patterns showed the broad nature of diffraction bands, indicating small particle sizes. These findings were in agreement with previous reports (47, 48). The sizes of designed nanoparticle formulations were around 200 to 300 nm in diameter, and PDI values were about 0.2 to 0.3, which is ideal for stability and uniformity of dispersion (49). The higher ZP of RHL-Fe_3_O_4_@PVA@*p*-CoA/GA NPs indicated good coating through Fe-O linkage with a repulsive negative charge barrier that helped avoid aggregation and improved the colloidal stability of RHL-Fe_3_O_4_@PVA@*p*-CoA/GA NPs. (50, 51).

TEM results showed the presence of the protective organic coat formed of GA and *p*-CoA that contributed to the previously reported data about the role of GA and other organic materials in reducing aggregate formation (52). In principle, RHL coatings promote a high superficial crystalline quality of hydrophobic magnetic nanoparticles. Furthermore, coating them with a lipid layer renders the solution hydrophilic and prevents its agglomeration (an aqueous suspension is necessary for bioapplications). Additionally, it provides accessible chemical moieties for bioconjugation (53). Also, this continuous release of *p*-CoA and GA from the RHL-Fe_3_O_4_@PVA NPs may be because at high pH values, the electrostatic association is moderately weak, and the lipid matrix was degraded (54).

A previous report stated that *p*-CoA has an inhibitory effect against various Gram-positive and Gram-negative bacteria and is less effective at selecting for resistance (55). A comparison between ZnO nanoparticles functionalized with GA and nonfunctionalized ZnO nanoparticles revealed the effectiveness of GA as having antibacterial activity against methicillin-resistant S. aureus and E. coli (56) in addition to its ability to suppress the biofilm formation of E. coli and S. aureus (57, 58). Additionally, rhamnolipid biosurfactants previously showed their ability to prevent biofilm formation by affecting the initial attachment onto various surfaces (59).

Synergistic antibacterial and antiadhesive properties of rhamnolipid-coated silver and iron oxide (Fe_3_O_4_) NPs against S. aureus biofilms were also confirmed previously (60). Corroborating these findings, here, we initially showed that antibacterial potency of RHL-Fe_3_O_4_ biosurfactant NPs against S. aureus and E. coli bacteria was strongly increased when conjugated with *p*-CoA and GA. Notably, RHL-Fe_3_O_4_@PVA@*p*-CoA/GA biosurfactant NPs exhibited a marked inhibitory effect of zone diameters up to 45 mm and MICs of 1 to 64 μg mL^−1^ against almost all of the tested strains. Furthermore, MBC values of all screened isolates indicating the bactericidal effect of RHL-Fe_3_O_4_@PVA@*p*-CoA/GA biosurfactant NPs were confirmed by close MBC and MIC values. Lou et al. mention that *p*-CoA effectively inhibited the growth of all tested bacterial pathogens, with MIC values of 20.64 μg mL^−1^ of S. aureus and 80.64 μg mL^−1^ of E. coli (55).

These results led us to investigate the mechanism of inhibition by SEM and TEM, which confirmed the synergistic effect of RHL-Fe_3_O_4_@PVA@*p*-CoA/GA biosurfactant NPs on the outer membrane of E. coli and S. aureus that may have resulted from the initial damage of the microbial lipid membrane by *p*-CoA and GA compounds. This observation is supported by previous studies that showed markedly disruptive activity of a laccase-catalyzed chitosan-GA derivative on E. coli and S. aureus cell membranes, causing leakage of cytoplasm (61).

Gallic acid is a phenolic acid present in numerous foods and is also an antimicrobial agent, affecting bacterial cell membranes and causing irreversible changes in permeability, rupture, and pore formation (62). *p*-CoA significantly increased the outer plasma membrane permeability, resulting in loss of barrier function and leakage of cytoplasmic contents (55). Moreover, the amphiphilic properties of RHL, owing to the presence of both hydrophilic and hydrophobic parts, allow it to interact with the nonpolar part of the cell membrane and penetrate the cell wall and plasma membrane; then, there is leakage of inner cytoplasmic materials to the exterior, leading to cell death (63). Allowing to cross Fe_3_O_4_ NPs inside the cell membrane and release GA and *p*-CoA along with Fe^2+^ ions into the periplasm and cytoplasm (64) increased the production of intracellular oxidative stress, which further augments the magnitude of damage and disruption to the membrane (44, 65).

Considering biofilm formation, the pathogenicity of S. aureus and E. coli is widely related to their ability to express surface proteins that are required for adhesion to host extracellular matrix molecules and their ability to persist through biofilm formation. It was demonstrated previously that rhamnolipids, in the concentration range from 10 to 200 μg mL^−1^, could prevent the attachment of E. coli, as well as Staphylococcus epidermidis, on glass, and they efficiently inhibited biofilm formation of two antibiotic-resistant MRSA strains on silicone catheter and glass. Our data investigate the impairment effect of RHL-Fe_3_O_4_@PVA@*p*-CoA/GA biosurfactant NPs on biofilm formation for E. coli serotypes and S. aureus strains; this may be related to the alteration of the surface hydrophobicity due to the presence of RHL shells on nanoparticles, preventing bacterial cell attachment to the surface (66).

The major constituents of staphylococcal biofilms are polysaccharides such as poly-*N-*acetylglucosamine (PNAG), which is synthesized by a single intercellular adhesion (*IcaABCD*) gene locus of four genes (13, 67). Additionally, important components of E. coli biofilms are curli fibrils that are encoded by the *CsgBAC* operon, which includes the *crl*, *csgA*, and *csgD* genes of E. coli (12). Thus, we investigated the transcriptional effect of RHL-Fe_3_O_4_@PVA@*p*-CoA/GA biosurfactant NPs on curli fibrils and polysaccharides as major important constituents of E. coli and staphylococcal biofilms, respectively. Here, we have shown that the highest downregulation in biofilm-associated *cs*g*A*, *csgD*, and *crl* genes was observed in E. coli O26:H11, and there was a significantly higher efficacy in downregulation of *icaA* and *icaD* genes of VRSA over the vancomycin antibiotic control. This finding may be related to previous studies that detected the ability of GA to suppress E. coli biofilm formation by regulating *pgaABCD* gene expression (57), and it had a specific antibiofilm effect on S. aureus by regulating the expression of the *ica* operon (58). New strategies for inhibiting biofilms are becoming increasingly necessary. Plant extracts and compounds are being explored as natural alternatives to existing synthetic antimicrobials (68).

Previous studies showed that GA exhibited the greatest inhibition activity on the growth (39.01%) and biofilm formation (60.23%) of 48-h cultures of E. coli at 25°C (69). Sharaf et al. demonstrate that Fe_3_O_4_ built into nanostructure lipid carriers (NLCs) has dual mechanisms of bactericidal activity and adhesion and has the ability to penetrate the bacterial cell membrane, resulting in production of reactive oxygen species (ROS), which, in turn, leads to damaged DNA, denaturation of protein, and inhibition of internal enzymes of six common pathogenic bacteria strains, ultimately leading to cell death (70). *p*-CoA may attach to the phosphate anion in the DNA double helix and intercalate the groove, thereby affecting replication, transcription, and expression (55). This finding needs further investigation to show the exact mechanisms of NPs binding with the expressed bacterial biofilm-associated proteins by using molecular docking and simulating its effect by using three-dimensional Swiss modeling.

**FIG 10:**
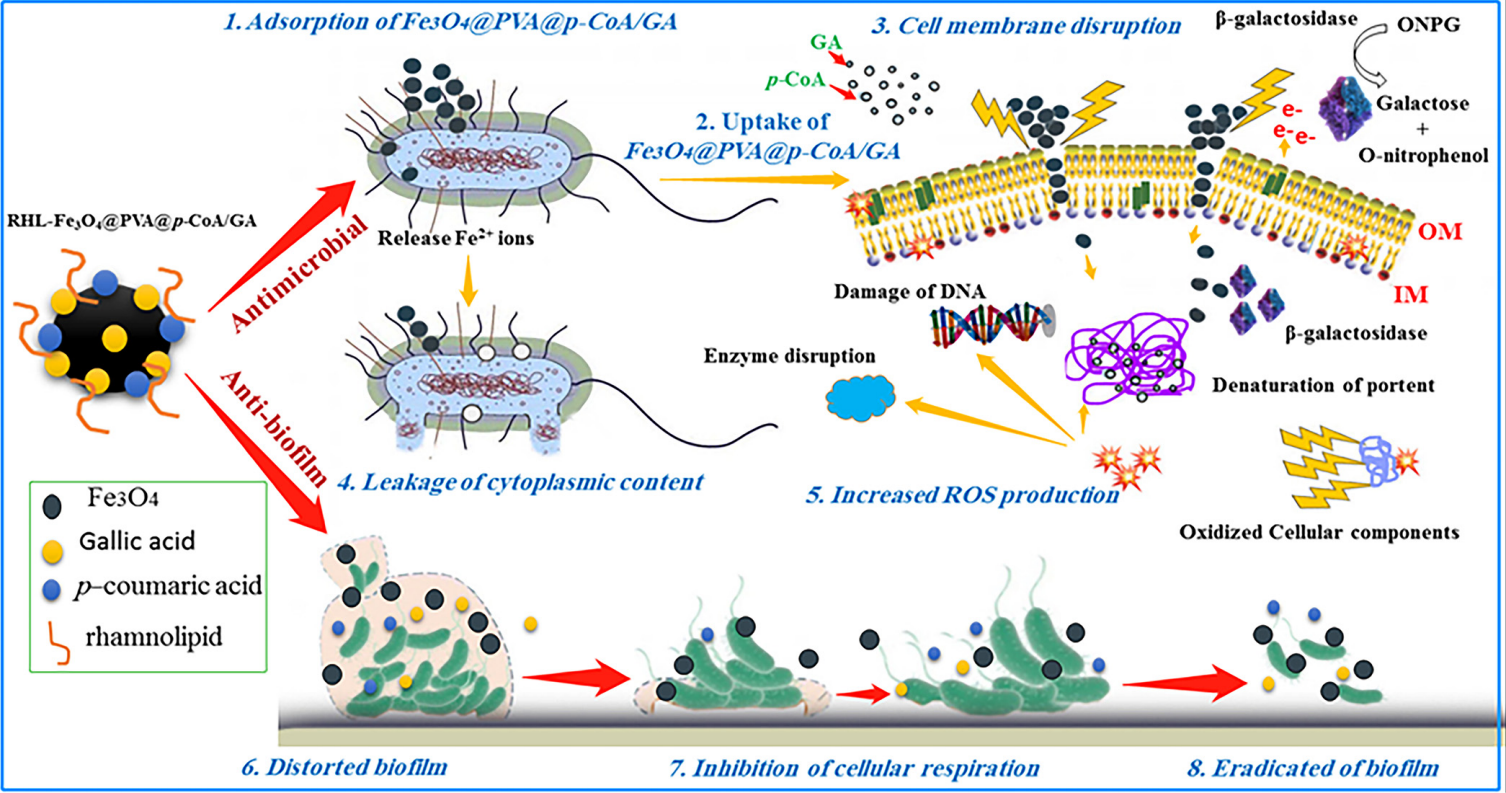
Proposed mechanistic illustration of multitarget activity of RHL-Fe_3_O_4_@PVA@*p*-CoA/GA biosurfactant NPs of suppression/inhibition on the plankton bacteria-biofilm interface and the activation of the bacterial cell death signaling cascade. ONPG, *o*-nitrophenyl-β-d-galactopyranoside.

In summary, the present study highlights the dual-target therapy of novel magnet biosurfactant NPs loaded with *p*-CoA and GA compounds, which improve bacterial treatment by inhibiting *in vitro* bacterial growth and targeting bacterial biofilm formation, consequently slowing the development of antibiotic resistance. First, antibacterial properties may result from initial damage of the microbial lipid membrane, and RHL amphiphilic properties interact with the cell membrane, allowing Fe_3_O_4_ NPs to cross inside and release GA and *p*-CoA, along with Fe^2+^ ions, into the cytoplasm, consequently activating the bacterial cell death signaling cascade (Fig. 10) (33). Second, antibiofilm properties are likely due to the direct binding and downregulation in biofilm-associated *IcaABCD* and *CsgBAC* gene loci (Fig. 10). Based on the above-described results, the synthesized RHL-Fe_3_O_4_@PVA@*p*-CoA/GA biosurfactant NPs have interesting properties, making them excellent candidates for targeted drug delivery.

The present study has some limitations, a few shortcomings that need to be explored further. These include, first, studying the *in vitro* effect of designed formulations on preestablished biofilms. Second, the lack of funds during the time of this study precluded us from doing a comparison of the designed formulations with standard bare Fe_3_O_4_, PVA, *p*-CoA, and GA, which are definitely the best approaches when it comes to predicting synergistic effects of different components. Obviously, future investigations will show that the combination of standard bare Fe_3_O_4_, PVA, *p*-CoA, and GA that provide the information needed to predict synergism and disruptive effect on a biofilm-based infection that already have established. In addition, we recommend undertaking cytotoxicity, along with *in vivo*, studies to demonstrate the efficacy of our designed formulations.

## MATERIALS AND METHODS

### Reagents.

FeCl_3_·6H_2_O and FeCl_2_·4H_2_O were obtained from Aladdin Chemical Reagent Company (Shanghai, China); *p*-coumaric acid, and gallic acid were purchased from Sigma-Aldrich (Germany); and Luria-Bertani (21) agar medium was purchased from Merck (Germany). Phosphate-buffered saline (PBS) was purchased from Solarbio Science and Technology (China). Rhamnolipids and glutaraldehyde were purchased from Sinopharm (Beijing, China). Phosphotungstic acid was purchased from PRA Health Sciences (Fort Washington, PA). All experiments were conducted using sterile deionized water (Milli-Q water).

### Bacterial strains and culture conditions.

Methicillin-susceptible S. aureus (MSSA; ATCC 25923), methicillin-resistant S. aureus (MRSA; ATCC 33592), vancomycin-resistant S. aureus (VRSA, ATCC 1001123), E. coli O157:H7 (ATCC 43888), E. coli O26:H11 (ATCC BAA-2196), and E. coli O78:H11 (ATCC H10407) were used in this study. S. aureus and E. coli were cultured in broth medium (21) at 37°C for 24 h with a 200-rpm continuous rotary shaker. Using UV-visible (UV-Vis) spectroscopy (Tu 1810; Beijing, China), the colonization was compiled and assembled in Hanks balanced salt solution (HBSS; pH 6.0) to an optical density at 550 nm (OD_550_) of 0.06, which corresponded to ~10^7^ CFU mL^−1^, and was then used in the following experiments.

### Synthesis of bare Fe_3_O_4_.

For simplicity and efficiency, the coprecipitation process for preparing bare Fe_3_O_4_ could be the most promising (71). FeCl_3_·6H_2_O and FeCl_2_·4H_2_O in a 2.75:1 molar ratio were dissolved in 150 mL of distilled deionized water (ddH_2_O) and heated at 75°C for 10 min. The iron solution was then rapidly added to 150 mL of 1.5 M NaOH and stirred for an hour at 800 rpm under the same temperature. Then, the nanoparticles were separated by using a powerful magnet and washed three times in deionized (DI) H_2_O and once in 0.01 M HCl. Finally, DI H_2_O was used to dilute the nanoparticle solution to a total volume of 50 mL.

### Preparation of PVA-coated Fe_3_O_4_.

The coating of PVA onto magnetic nanoparticles was achieved according to the emulsion-coacervation method (72) with some modifications. Twenty milliliters of uncoated Fe_3_O_4_ were prepared as described above and capped with PVA by adding an equal volume of 2% (wt/vol) PVA solution under strong magnetic stirring at 800 rpm for 12 h at 20 to 25°C (pH 7.2). Then, the final formula of PVA@Fe_3_O_4_ was washed four times with DI H_2_O to remove all excess noncoated PVA polymers, and finally, the PVA@Fe_3_O_4_ was collected using a strong permanent magnet and dried in an oven at 40°C overnight.

### *p*-CoA and GA loading of PVA@Fe_3_O_4_.

The purified PVA-coated colloidal magnetic nanoparticles were redisposed in DI H_2_O and left at room temperature. We dissolved 0.2% (wt/vol) of pure GA and 0.3% (wt/vol) of pure *p-*CoA in ddH_2_O with shaking for 10 min. We added 20 mL of GA and *p-*CoA solution to the PVA@Fe_3_O_4_ solution, and the mixture was vigorously stirred for 15 min at 20 to 25°C. Then, Fe_3_O_4_@PVA@*p*-CoA/GA NPs were aggregated to remove all extra drugs by centrifugation at 7,000 rpm for 15 min several times with additional ddH_2_O each time. Finally, Fe_3_O_4_@PVA@*p*-CoA/GA NPs were collected by a strong magnet and dried in a vacuum for 12 h at 60 to 70°C.

### RHL core cell coating onto Fe_3_O_4_@PVA@*p*-CoA/GA NPs.

Ten milliliters of Fe_3_O_4_ were stirred continuously for 3 h at room temperature with a few drops of 2 M HNO_3_. The sample was thoroughly washed with Milli-Q water. The pH of the HNO_3_-treated Fe_3_O_4_ nanoparticles was adjusted to pH 5.5. Fifty milliliters per mole of RHL were dispersed into 10 mL of NaCO_3_ (0.03% [wt/vol]) and used to coat Fe_3_O_4_ NPs at a ratio of 1:1 (vol/vol) for 48 h at 37°C and then centrifuged at 2,500 rpm for 1 min. Finally, the samples were washed with ddH_2_O and stored at 4°C for description.

### Characterization of RHL-Fe_3_O_4_@PVA@*p*-CoA/GA biosurfactant NPs.

FTIR spectra measurements were determined using a PerkinElmer 2000 spectrophotometer. Each sample was mixed with crystalline KBr in a 1:10 (sample/KBr) ratio and left for 6 min on the disk for dehydration. A spectrum was measured using wavenumbers in the 400 to 4,000 cm^−1^ range. X-ray diffraction (XRD) patterns for Fe_3_O_4_- and PVA@Fe_3_O_4_-loaded *p-*CoA and GA were obtained with a Philips PW 1710 X-ray diffractometer using Cu Kα radiation (λ = 1.78987 Å) at the ranges angle (2θ) 12 in the range of 5° to 80°, and the tube was operated at 40 kV and 30 mA. The vibrating sample magnetometer (VSM) was examined with a 0.5 T physical property measurement system (PPMS-9; Quantum Design, USA) at 300 K.

### RHL-Fe_3_O_4_@PVA@*p*-CoA/GA biosurfactant NP dispersions.

The means of PS and ZP for the formulations were measured by dynamic light scattering (DLS) using (Malvern Instruments, UK). Each sample was diluted in 3 mL of DI H_2_O and placed in a different cell cuvette; the average size was obtained after three measurements, and zeta potential was measured four times. TEM analysis was performed using a Hitachi H-7100 (Tokyo, Japan; 90 and 200 KV) after making dilutions of Fe_3_O_4_ and Fe_3_O_4_@PVA@*p*-CoA/GA and RHL-Fe_3_O_4_@PVA@*p*-CoA/GA biosurfactant NP samples with ddH_2_O at a ratio of 1:1,000 μL/mL^−1^. Then, a one-drop putting of each dilution nanoparticle solution into carbon-coated copper grids. Afterward, the deposited samples were allowed to dry for 5 to 10 min. SEM was used to observe the surface morphology of the samples using a JSM-6400 machine (Joel, Tokyo, Japan) (48).

### Determination of antimicrobial proprieties (MICs) using agar well diffusion assay and broth microdilution assays.

**(i) Agar well assay.** RHL-Fe_3_O_4_@PVA@*p*-CoA/GA biosurfactant NPs were screened for activity against all bacterial isolates using an agar well diffusion assay (73). Bacterial suspensions in sterile saline corresponding to an optical density of a 0.5 McFarland standard (1.5 × 10^8^ CFU/mL) were grown in Mueller-Hinton (MH) agar (Oxoid Ltd., England). Wells (8 mm) were cut into each inoculated agar plate, and a 100-μL aliquot of each NP solution (10 μg/mL concentration) was pipetted into each well. NPs were replaced with sterile water as a negative control for bacterial growth and standard antimicrobial discs (Oxoid, Cambridge, UK). Imipenem (IPM; 10 μg) and ciprofloxacin (CIP; 5 μg) were used as positive control for bactericidal action for E. coli, while vancomycin (VA; 30 μg), ciprofloxacin (CIP; 5 μg), and oxacillin (OX; 1 μg) were used for S. aureus. The plates were incubated at 37°C for 24 h. After incubation, zones of growth inhibition were measured to the nearest millimeter to determine the antimicrobial potency of the screened antimicrobial substances (74). The results are expressed as mean ± standard deviation (75).

### (ii) MIC measurements.

The MICs of Fe_3_O_4_@PVA@*p*-CoA/GA NPs and RHL-Fe_3_O_4_@PVA@*p*-CoA/GA biosurfactant NP suspensions were determined according to CLSI guidelines (76). Briefly, the antibiotic controls, RHL-Fe_3_O_4_, and RHL-Fe_3_O_4_@PVA@*p*-CoA/GA were serially diluted 2-fold across a 96-well tray with a range of concentrations from 0.125 to 256 μg/mL^−1^ (77), and the wells were inoculated with 1 × 10^6^ CFU/mL of bacteria and then incubated for at 37°C for 24 h. The positive control was created without adding any nanoparticles or antibiotics, and the negative control was made without inoculating bacteria. MICs for all organisms were determined visually using a reading mirror as the lowest concentration of product preventing growth and resulting in no turbidity. The minimum bactericidal concentrations (MBCs) were determined by subculturing 10-μL aliquots from nonturbid wells onto blood agar. After incubating the agar plates, colonies were counted, and the lowest concentration showing a 99.9% reduction in the initial inoculum was recorded as the MBC. The subinhibitory concentrations (sub-MICs) were determined as 0.5× MICs (78).

### Scanning electron microscopy analysis.

E. coli O78:H10 and MRSA were treated with MICs of RHL-Fe_3_O_4_@PVA@*p*-CoA/GA biosurfactant NP solutions and incubated at 34 ± 3°C for 3 and 12 h. Then, bacterial cells were centrifuged at 3,500 rpm for 3 min. In addition, they were washed in 100 mM PBS, pH 7.4. Afterward, the bacterial cell pellets were blended for 2 h at 4°C with 500 μL of 2.5% (vol/vol) glutaraldehyde. The specimens were then dehydrated in various concentrations of ethyl alcohol (30% to 100%) for 10 min before being washed with PBS. After being washed twice in a 1:1 ethanol-butanol solution and dispersed in 100% pure butanol, the samples were dehydrated to critical levels. Finally, a 200-Å Au film was applied to the bacterial samples, and a micrograph was taken with an SEM at 10-kV acceleration voltages for analysis (JSM6510LV; Japan) (79).

### Transmission electron microscopy analysis.

A drop of the E. coli O78:H10 bacterial suspension was layered on a form var-coated copper grid with a micropipette and left for 2 min at 25°C (or room temperature) to allow the bacteria to bind to the copper grid. After that, a 1% decrease in phosphotungstic acid (PTA; Sciences, Fort Washington, PA) was applied. Finally, the grid was incubated for 2 min before being visualized by TEM at 24-h intervals (80).

### Antiadhesive properties of RHL-Fe_3_O_4_@PVA@pCoA/GA biosurfactant NPs and biofilm formation assay.

**(i) Crystal violet assay.** Bacterial strains were inoculated in 96-well polystyrene microtiter plates at approximately 10^6^ CFU/mL in Mueller-Hinton broth with subinhibitory concentrations (0.5× the MICs) of either RHL-Fe_3_O_4_, RHL-Fe_3_O_4_@PVA@*p*-CoA/GA, or antibiotic controls. The antibiotic-free medium in well was used as negative control. Then, the plates were incubated at 37°C overnight to allow the biofilm formation, and the quantification of biofilms was performed by using the crystal violet assay; the optical density of the biofilm was measured by a microplate enzyme-linked immunosorbent assay (ELISA) reader (HumaReader HS, Germany) at a wavelength of 570 nm as described previously (81). The experiment was performed in triplicate separately for each strain, the average value was calculated, and biofilm formation was described as the ratio of OD_570_ of the sub-MICs of RHL-Fe_3_O_4_, RHL-Fe_3_O_4_@PVA@*p*-CoA/GA, or antibiotic controls to the OD_570_ of untreated negative control (82, 83).

### (ii) Quantitative real-time PCR assay for biofilm-related S. aureus and E. coli genes.

qRT-PCR was carried out with bacterial isolates grown in the presence of subinhibitory concentrations of either NPs or antibiotic control as described in an earlier section (crystal violet assay) with untreated negative control as reference, using triplicates for each condition. Then, RNA extraction was performed using QIAamp RNeasy minikit (Qiagen GmbH, Germany) according to the manufacturer’s instructions. Genomic DNA was removed from the samples by treatment with 1 U DNase I, RNase-free (Thermo Scientific) for 60 min at 37°C. Real-time PCR amplification reaction mixtures were prepared in a final volume of 25 μL containing 10 μL of 2× Hera SYBR Green RT-qPCR master mix (Willowfort, UK), 1 μL of RT enzyme mix (20×), 0.5 μL of each primer of 20 pmol concentration, 5 μL of RNase- and DNase-free water, and 3 μL of RNA template.

The primer sequences used for the genes involved in biofilm formation for S. aureus (*icaA* and *icaD*) and E. coli (*csgA*, *csgD*, and *crl*) are shown in Table 2. The 16S rRNA gene was used as an internal control for the normalization of the mRNA expression. The reaction was performed using a StepOne real-time PCR system (Applied Biosystems, CA, USA). The relative expression fold changes of mRNAs were calculated using the threshold cycle (2^−ΔΔ^*^CT^*) method. The relative expression of each gene after the exposure of the bacteria to sub-MICs of NPs and antibiotic controls was normalized to the untreated negative control, which was assigned a value of 1 arbitrary unit.

**TABLE 2:** Primer sequences and target genes of S. aureus and E. coli

Bacterial isolate	Target gene	Primer sequence	Reference
E. coli	16S RNA	GACCTCGGTTTAGTTCACAGA	77
		CACACGCTGACGCTGACCA	77
	*csgA*	CGGAGTGGATGTTAACGACTGG	38
		ATGTTCGCAGACCCAGTCATTG	38
	*Crl*	GCATCTGGGAAGGAACTAGGG	38
		TGAACCACAAGCATAGCCCA	38
	*csgD*	CAAGAGGAAAACTCCAGTAATTGCA	78
		AAGTCGAAGAGGAAGGCCATAA	78
S. aureus	16S RNA	CCTATAAGACTGGGATAACTTCGGG	79
		CTTTGAGTTTCAACCTTGCGGTCG	79
	*icaA*	CCT AAC TAA CGA AAG GTA G	80
		AAG ATA TAG CGATAA GTG C	80
	*icaD*	ATGGTCAAGCCCAGACAGAG	80
		AGTATTTTCAATGTTTAAAGCAA	80

### Statistical analysis.

Each experiment was carried out at least in triplicate, and all data were presented as mean ± standard deviation (SD). Analysis of statistical significance was performed by one-way analysis of variance (ANOVA) and the *post hoc* Tukey test (*P* < 0.05). All analysis was conducted in SAS 9.4 for Windows 64-bit from SAS Institute (Cary, NC), and graphical outputs were generated by GraphPad Prism software (version 8; GraphPad Software Inc.).
