# Supplementary material for: Rhamnolipid-Coated Iron Oxide Nanoparticles as a Novel Multitarget Candidate against Major Foodborne E. coli Serotypes and Methicillin-Resistant S. aureus
Source: Microbiol Spectr. 2022 Jul 19;10(4):e00250-22. doi: 10.1128/spectrum.00250-22 (PMC9430161; doi:10.1128/spectrum.00250-22)
Supplement: Supplemental file 1 — Supplemental material. Download spectrum.00250-22-s0001.pdf, PDF file, 0.5 MB [file spectrum.00250-22-s0001.pdf]

Supporting Information

Rhamnolipids coated iron oxide nanoparticles as a novel multi-target candidate against major foodborne *E. coli* serotypes and methicillin-resistant *S. aureus*

Mohamed Sharaf[1, 2♣], Alaa H. Sewid[3, 4], H. I. Hamouda[5, 6], Mohamed G. Elharrif[7], Azza S. El-Demerdash[8], Afaf Alharthi[9], Nada Hashim[10♣], Samy Selim[11], Anas Abdullah Hamad[12], Dalal Hussien M. Alkhalifah[13], Wael N. Hozzein[14], Mohnad Abdalla[15♣] and Taisir Saber[16]

♣Corresponding author: *E-mail address*: mohamedkamel@azhar.edu.eg(M.S), med@uofg.edu.sd(N.H.) and mohnadabdalla200@gmail.com(M.A.)

Table S1.Average Size, PDI and zeta potential *p*-CoA and GA loading efficiency in the prepared nanoparticles

**Sample**	**Size (nm)**	**PDI**	**zeta potential (mV)**	**Leading efficiency (%) (*p*-CoA)**	**Leading efficiency (%) (GA)**
**Fe_3O_4**	15.09±2.8	0.385±0.16	−18.7±0.22	–	–
**RHL-Fe_3O_4**	25±4.8	0.153±0.04	−33.3±0.66	–	
**Fe_3O_4@PVA@*p*–CoA/GA**	213.8±26.6	0.264±0.017	−33.7±0.25	95±2.8	97±2.2
**RHL–Fe_3O_4@PVA@*p*–CoA/GA**	254.6±32.1	0.202±0.014	−34.3±0.33	–	–

Numerical data are reported as mean ± SD to Z-potential (*n*=3) and particle size and PDI (*n*=4).

A

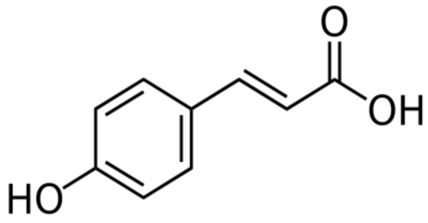

B

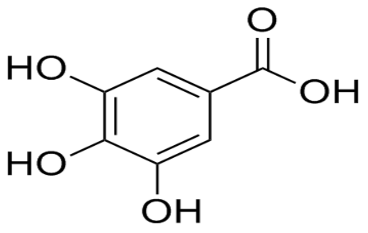

Fig.S1**:** Chemical structures of (A) *p*-Coumaric acid (*p*-CoA), and (B) Gallic acid (GA)

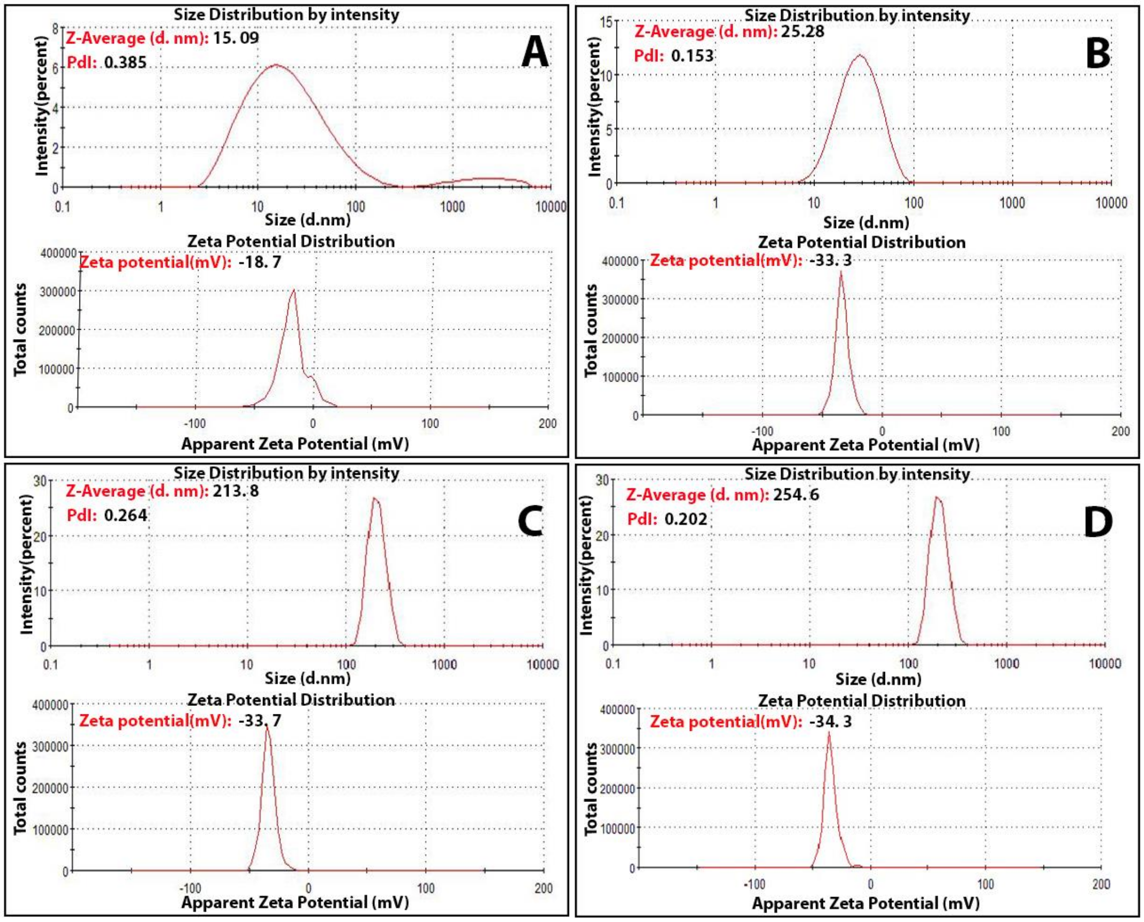

Fig.S2 Hydrodynamic size, zeta potential and polydispersity index (PDI) to different Fe_3O_4 formulation; (A) bare Fe_3O_4, (B) Fe_3O_4@PVA, (C) Fe_3O_4@PVA@*p*–CoA/GA NPsand (D)RHL–Fe_3O_4@PVA@*p*–CoA/GA biosurfactantsNPs. Numerical data is reported as mean ± SD z-potential (*n*=3) and particle size and PDI (*n*=4).
